# Modelling Excess Public Expenditure on Emergency Department and Inpatient Care Associated with Gambling Disorder in Spain

**DOI:** 10.3390/healthcare14213685

**Published:** 2026-10-31

**Authors:** Gonzalo Romero-Martín, Rosa Magallón Botaya, Ana Gastón Faci, Eva María Andrés Esteban

**Affiliations:** 1 Facultad de Emprendimiento, Empresa y Ciencias Jurídicas, Universidad Camilo José Cela, C. de Juan Hurtado de Mendoza 4, 28036 Madrid, Spain; 2 Facultad de Economía y Empresa, Universidad Internacional de La Rioja, Av. de la Paz 137, 26006 Logroño, Spain; 3 Departamento de Medicina, Psiquiatría y Dermatología, Facultad de Medicina, Universidad de Zaragoza, C. Domingo Miral s/n, 50009 Zaragoza, Spain; 4 Red de Investigación en Actividades Preventivas y Promoción de la Salud (redIAPP), Instituto de Salud Pública Carlos III, C. Sinesio Delgado 4, 28029 Madrid, Spain; 5 GIIS011, IIS Aragón ( Instituto de Investigación Sanitaria Aragón), Av. San Juan Bosco 13, 50009 Zaragoza, Spain; 6 Departamento de Economía Aplicada II, Facultad de Economía y Empresa, Universidad Rey Juan Carlos, Calle Tulipán, Móstoles, 28933 Madrid, Spain

**Keywords:** gambling disorder, cost of illness, public expenditure, healthcare costs, economic burden, public health

## Abstract

**Highlights:**

**What are the main findings?**

- Under the stated transport assumptions, gambling disorder was associated with an estimated EUR 346.32 million in annual excess emergency and inpatient expenditure in Spain, equivalent to EUR 1354.17 per adult with GD.
- Hospitalisation accounted for 98.1% of the excess expenditure estimated for the two included services; this proportion does not describe the overall healthcare burden of GD.

**What are the implications of the main findings?**

- The estimates identify data gaps relevant to healthcare planning, particularly the need for Spanish evidence on inpatient use among adults with GD.
- The model did not evaluate prevention or treatment effects and cannot quantify avoidable hospitalisations or achievable healthcare savings.

**Abstract:**

**Background:** Evidence on healthcare expenditure associated with gambling disorder (GD) remains limited in Spain. This study modelled annual excess emergency department and inpatient expenditure per adult with GD and nationally. **Methods:** A prevalence-based cost model applied international adjusted healthcare-use odds ratios to Spanish baseline probabilities, using a one-episode approximation and national DSM-5 GD prevalence. Hospitalisation was valued at the provisional 2024 SNS mean (EUR 6004.93 per discharge); emergency attendance used the EUR 185 Madrid tariff introduced in December 2023. Deterministic and restricted probabilistic analyses examined uncertainty; additional scenarios examined source choice, episode intensity and overlap. **Results:** The updated base-case estimate was EUR 1354.17 per adult with GD and EUR 346.32 million nationally; hospitalisation contributed 98.1% of this two-service total. Varying its odds ratio across the reported confidence interval gave EUR 109.67–EUR 691.76 million. Using an alternative adjusted inpatient association from the emergency-use source reduced the total to EUR 44.60 million, demonstrating sensitivity to source selection. **Conclusions:** The model estimates selected excess emergency and inpatient expenditure associated with GD under explicit assumptions. Diagnostic, comparator and outcome differences limit transferability to Spain. These estimates are neither a validated lower bound nor evidence of causally attributable expenditure or intervention savings.

## 1. Introduction

Gambling is increasingly recognised as a major public health challenge rather than solely a matter of individual responsibility or recreational behaviour. The rapid expansion of online gambling, mobile betting platforms and continuous-access gambling environments has substantially increased exposure to potentially addictive gambling products. Consequently, international organisations and public health experts now advocate addressing gambling as a commercial determinant of health requiring population-based prevention strategies comparable to those implemented for tobacco and alcohol [1,2].

Although most individuals who gamble do not develop GD, gambling-related harm extends beyond clinically diagnosed cases. Clinical evidence links GD with depression, anxiety, substance-use disorders and suicidal behaviour, as well as financial and social difficulties [3]. Spanish adolescent research has separately identified clustered addictive behaviours and socioeconomic vulnerability [4]; it is not used here as evidence for adult psychiatric outcomes. Healthcare use may reflect both gambling-related harms and co-occurring conditions [1,5,6].

The principal utilisation sources also differ substantially. Morasco et al. analysed US survey data using lifetime DSM-IV pathological gambling and past-year service use [5]. Bhattacharjee et al. modelled inpatient use in English APMS data and compared fiscal consequences across gambling-risk groups [6]. Applying these associations to current DSM-5 GD in Spain requires assumptions about clinical severity, comparator risk and service definitions; the model therefore estimates conditional excess expenditure rather than an observed Spanish causal effect.

Reliable estimates of healthcare expenditure associated with GD are essential for healthcare planning and resource allocation. However, previous cost-of-illness studies have reported widely differing estimates because of variation in analytical perspectives, attribution methods and cost components. Most studies have adopted a broad societal perspective, combining healthcare expenditure with productivity losses, criminal justice costs and other indirect consequences, limiting comparability across countries [7,8,9]. More recently, some studies demonstrated that estimates are highly sensitive to the attribution method used, highlighting the need for transparent and reproducible costing approaches [9,10].

Recent evidence further emphasises the importance of methodological transparency. A 2025 scoping review identified substantial variation in the cost categories and methods used to estimate the social costs of gambling [11]. In February 2026, Moore et al. reported associations between gambling frequency and harm severity in England and Scotland, providing evidence relevant to future public health economic models [12]. These studies support distinguishing observed associations from causal effects and specifying which outcomes a cost model includes.

Spain provides a particularly relevant setting for this analysis. A recent nationally representative survey estimated a prevalence of gambling disorder of 0.63% among adults [13], while the regulated online gambling market continues to expand rapidly [14,15]. Because Spain operates a universal publicly funded healthcare system, a substantial proportion of the healthcare consequences associated with GD are likely to be borne by the National Health System. From a health economics perspective, quantifying the economic burden associated with disease can provide relevant evidence for healthcare planning and resource allocation, as illustrated by recent cost-of-illness research in the Spanish context [16]. In the case of gambling disorder, the absence of such evidence limits the ability of policymakers to assess its budgetary implications and the potential value of prevention and treatment strategies relative to the healthcare expenditure associated with the disorder.

Recent national surveys illustrate the extent of gambling across European Union countries. Past-year gambling participation was 36.4% among people aged 16–70 years in Germany in 2025 [17], 51.6% among those aged 18–75 years in France in 2023 [18], and 70% among those aged 15–74 years in mainland Finland in 2023 [19]. Participation should be distinguished from gambling disorder: the German survey estimated DSM-5 gambling disorder in 2.2% of adults aged 18–70 years (95% CI 1.9–2.5%) [17]. These figures provide context for the Spanish estimate of 0.63% [13]; however, differences in age coverage, survey design, assessment instruments and reference periods preclude interpreting them as a harmonised ranking of national prevalence.

This study aimed to model annual excess public expenditure on emergency department and inpatient care associated with GD in Spain, expressed per adult with GD and nationally. The focus is limited to these two services.

## 2. Materials and Methods

### 2.1. Study Design

This prevalence-based cost-of-illness study adopted the perspective of the Spanish National Health System (*Sistema Nacional de Salud*, SNS) and a one-year time horizon. The costing reference year was 2024. Only direct publicly funded healthcare expenditure was considered; productivity losses, criminal justice, social care, informal care, private expenditure and intangible harms were outside this perspective.

A prevalence-based approach was selected to estimate costs associated with existing GD over one year rather than lifetime costs of incident cases. This distinction follows the methodological description of prevalence- and incidence-based cost-of-illness approaches [20]; it is not presented as a universal recommendation for resource allocation.

Reporting was informed by applicable CHEERS 2022 items on perspective, time horizon, data sources, valuation, modelling and uncertainty [21]. CHEERS is a reporting framework, not evidence that model assumptions are valid. STROBE principles were used only to guide discussion of the definitions, confounding and limitations of the observational input studies [22]; the present analysis is a secondary model rather than a newly conducted cohort or cross-sectional study.

### 2.2. Analytical Framework

Candidate healthcare components were assessed for evidence on annual excess utilisation, availability of Spanish healthcare cost inputs and overlap with other services. Emergency department use and hospitalisation were included in the base-case calculation. Table 1 summarises the inclusion decisions. Primary care and gambling-specific treatment were considered potential extensions because their utilisation or costing inputs remained uncertain; no additional component estimates are reported here.

The target population comprised residents of Spain aged 18 years or older meeting at least four DSM-5 criteria for GD. The base-case prevalence was 0.63%. The estimated number of adults with GD in Spain was calculated as:

(1) $$N_{GD}=P_{adult}\times Prev_{GD}$$

where:

• $N_{GD}$ = estimated number of adults with gambling disorder in Spain;
• $P_{adult}$ = adult resident population (≥18 years) in Spain during the reference year;
• $Prev_{GD}$ = prevalence of gambling disorder in the adult population.

The base case used GD prevalence of 0.63% in the general adult population [13]. This prevalence was held fixed in the revised probabilistic analysis because no comparable population-based uncertainty interval was established. Deterministic sensitivity analysis used an explicitly assumed ±20% relative variation (0.504–0.756%), not an epidemiological confidence interval. The 1.05% estimate among past-year gamblers was removed from the national sensitivity analysis and probabilistic distribution because its denominator differs from the target population.

### 2.3. Conceptual Framework and Healthcare Cost Categories

Candidate components and inclusion decisions are reported in Table 1. Inclusion required a published healthcare-use association, an identifiable service and a Spanish valuation input, with explicit consideration of overlap. Emergency and inpatient estimates were retained as conditional proxies, rather than as validated measurements of annual GD-attributable use. Primary care and gambling-specific treatment remain potential extensions without numerical estimates.

Evidence was selected through a targeted, non-systematic assessment of relevant publications and official sources. For this revision, web-assisted searches on 20 October 2026 targeted PubMed-indexed records, publisher pages and the NIESR report, with combinations of gambling, gambling disorder, healthcare utilisation, hospitalisation, medical utilisation, cost-of-illness and Spain; reference lists and the INE, Ministry of Health and Comunidad de Madrid portals were checked. The purpose was source verification and identification of alternative inputs, not an exhaustive systematic review. Search terms and source links are documented in Supplementary File S1.

Eligible utilisation evidence concerned adults, identifiable healthcare contacts and an extractable adjusted association with uncertainty bounds. Where a study reported several models, the estimate with demographic and behavioural or clinical adjustment was preferred; statistical significance was not an eligibility criterion. The retained inpatient estimate was not pooled with the emergency-use study because definitions and comparators differ. Its selection is examined explicitly using an alternative within-study inpatient association [5,6]. No claim is made that the selected inputs are uniquely preferable or directly interchangeable.

#### 2.3.1. Estimation of Excess Healthcare Utilisation

For service j, modelled excess annual use was represented by the difference between a transported probability of any use and the Spanish baseline probability. The conversion in Equation (2) assumes that the published adjusted odds ratio can be applied to that baseline. It is a modelling transport assumption, not a causal risk difference estimated in Spain. Psychiatric comorbidity and socioeconomic or behavioural factors may explain part of the association.

The emergency department OR was 1.98 (95% CI 1.27–3.09), comparing lifetime DSM-IV pathological gambling with low-risk individuals in the US NESARC survey [5]. The retained inpatient OR was 5.575 (95% CI 2.14–14.50), reported for problem gambling in the APMS-based analysis [6]. That report separates gambling-risk groups and uses at-risk gambling in its fiscal counterfactual; the reported coefficient is not a directly validated DSM-5 GD-versus-Spanish-general-population contrast. Baseline probabilities of 0.23 for emergency use and 0.068 for hospitalisation were retained from Spanish aggregate statistics [23].

Transport therefore assumes comparable relative odds across countries, diagnostic definitions and comparator risks. Lifetime DSM-IV pathological gambling, DSM-IV problem gambling and current DSM-5 GD identify overlapping but non-equivalent groups, so the direction of transfer bias is uncertain. An alternative scenario used the fully adjusted inpatient OR of 1.40 (95% CI 0.85–2.29) for overnight hospitalisation in the same NESARC study as the emergency-use estimate [5]. This estimate was included despite its interval crossing one.

Spanish hospitalisation covers an overnight stay or an assigned bed during the preceding 12 months, excluding delivery or caesarean section [24]. The UK APMS documentation distinguishes annual and quarterly stays and includes private facilities while excluding childbirth [25]. These definitions provide partial clinical alignment, but the NIESR table does not establish that its specific inpatient variable matches the Spanish annual measure in all respects. Recall period, financing and case-mix differences remain unresolved; the model does not assume that a similar outcome label proves equivalence.

The original calculation retained the published aggregate baseline probabilities of 0.23 for emergency-service use and 0.068 for hospitalisation. To examine the implications of age coverage and population composition, we additionally analysed anonymised ESdE 2023 adult microdata, restricting the sample to respondents aged ≥18 years with positive survey weights (n = 20,639). The adult-specific weighted annual probabilities were 0.227354 for emergency-service use and 0.069578 for hospitalisation, excluding childbirth or caesarean section. These probabilities were used in a supplementary aggregate recalculation and in stratified sensitivity analyses incorporating age, sex, region and, in a separate scenario, education, as described in Section 2.3.3. The original aggregate-input calculation was retained as the reference analysis. Neither the survey-wide nor the stratum-specific probabilities identify utilisation specifically among adults without GD; their use as comparator probabilities remains a modelling assumption.

When adjusted odds ratios were reported, the probability of healthcare utilisation among adults with GD was estimated as:

(2) $$P_{GD,j}=\frac{OR_{j}\times P_{0,j}}{1-P_{0,j}+OR_{j}\times P_{0,j}}$$

where:

• $P_{GD,j}$ = estimated probability of healthcare utilisation among adults with GD;
• $OR_{j}$ = adjusted odds ratio;
• $P_{0,j}$ = baseline probability of healthcare utilisation in the Spanish adult population.

The excess probability was obtained by subtracting the baseline from the probability in Equation (2). For emergency use, 0.371634 − 0.23 = 0.141634; for inpatient use, 0.289147 − 0.068 = 0.221147. Multiplication by EUR 185 and EUR 6004.93 gives EUR 26.20 and EUR 1327.97 per adult with GD, respectively. These are modelled population averages, not episode frequencies or individual bills. Equation (1) gives the GD population and Equation (3) sums the service-level cost estimates.

The inpatient input was updated to EUR 6004.93, the provisional 2024 national mean cost per acute hospital discharge [26]. The emergency input of EUR 185 is the general non-admitted emergency tariff in Order 1975/2023, published on 30 December 2023 and effective the following day, and was retained as the identified tariff applicable at the start of 2024 [27]. It is a regional reimbursement proxy, not a nationally representative estimate of economic resource cost. Although tariff construction uses cost-accounting information, tariff and resource cost are conceptually different. No regional weighted average was calculated. The model combines the 2024 hospital cost with this tariff and does not claim fully harmonised national 2024 resource prices.

#### 2.3.2. Cost Calculations

Annual average excess healthcare cost per adult with GD. The annual average excess cost per adult with GD was obtained by summing the products of excess use and the corresponding healthcare cost input:

(3) $$C_{patient}=\sum_{j=1}^{k}Q_{j}\times UC_{j}$$

where:

• $C_{patient}$ = annual excess healthcare cost per adult with GD;
• $Q_{j}$ = excess annual utilisation of healthcare resource *j*;
• $UC_{j}$ = Spanish unit cost.

*k* denotes the number of included components. These averages use the entire population with GD as the denominator, including adults who did not use either service. Their sum does not imply that every adult attended an emergency department and was hospitalised.

National annual expenditure was calculated by multiplying the average excess cost per adult with GD by the estimated number of adults with GD. Results are reported by component and in total, alongside each component’s percentage contribution. No discounting was applied because the time horizon was one year.

The emergency tariff applies to attendances without admission, but the utilisation OR does not identify this subgroup. Residual double counting may therefore increase the combined estimate if costed emergency resources are also covered by inpatient costs. The main calculation retains both components conditionally; a structural scenario removes the entire costed emergency component to illustrate the maximum reduction from that component alone. This does not resolve broader comparator, attribution or diagnostic uncertainty.

For recurrent use, a single-event probability does not identify the expected number of episodes. If the excess episode count were 1.5 or 2 times the excess probability for each service, the corresponding cost would be multiplied by 1.5 or 2. These explicitly assumed intensity scenarios illustrate numerical consequences; they do not estimate recurrence, and neither they nor omitted services establish a lower bound. Different recurrence patterns between GD and comparator groups could change the excess in either direction.

#### 2.3.3. Parameter Selection, Validation and Sensitivity Analysis

The calculation combined Spanish population, baseline utilisation and costing inputs with internationally adjusted associations for healthcare use. Parameter values and sensitivity ranges are presented in Table 2. Missing evidence for an excluded component was not interpreted as evidence of zero cost.

Arithmetic consistency was assessed by comparing the component estimates, their sum and the population extrapolation. Such checks assess implementation and rounding, not the validity of causal attribution or parameter transferability. Repeating the same deterministic calculation with unchanged inputs would be expected to give the same result.

International studies were used to contextualise the findings in the Discussion. Differences in perspective, populations and included services preclude treating agreement with their totals as external validation.

One-way analyses varied ORs across their published confidence intervals and varied costs, baseline probabilities and adult GD prevalence by ±20%. The latter ranges are analyst-selected scenarios, not empirical 95% intervals. Table 2 reports all base inputs, including the baseline probabilities. Structural scenarios assessed source choice, episode intensity and complete omission of the costed emergency component. The restricted PSA in Appendix A varies only the two ORs; all other inputs are fixed.

Appendix A reports the revised restricted PSA, its distribution parameters, assumptions and results. It measures conditional OR uncertainty and does not quantify all model uncertainty.

An exploratory population-weighted stratified sensitivity analysis was conducted using anonymised microdata from the Spanish Health Survey (ESdE) 2023 [23,24]. The sample was restricted to respondents aged ≥18 years with positive adult survey weights (n = 20,639). Weighted annual probabilities of emergency-service use and hospitalisation, excluding childbirth or caesarean section, were estimated within 114 strata defined by region (19 categories), sex and age group (18–34, 35–64 and ≥65 years). The original OR-to-probability transformation was applied within each stratum, and excess costs were aggregated using normalised adult survey weights. The original service-specific ORs, unit costs, national adult population and GD prevalence were retained. Additional scenarios used age–sex strata and age–sex–education strata; education was grouped into lower, intermediate and university levels. All scenarios assumed constant GD prevalence and common transported ORs across strata. These analyses recalibrated baseline utilisation for population composition rather than estimating a new confounder-adjusted association between GD and healthcare use.

### 2.4. Data Sources and Processing

This study combined published estimates, publicly available aggregate statistics and a supplementary analysis of anonymised public-use microdata. No primary participant data were collected, and no identifiable individual-level information was accessed. The Instituto Nacional de Estadística (INE) provided the Spanish population statistics and, jointly with the Ministry of Health, the ESdE 2023 healthcare-use data [23,24,28]. The Comunidad de Madrid provided the emergency-care tariff [27], and the Ministry of Health provided the national hospital cost estimates [26]. GD prevalence and adjusted healthcare-use associations were extracted from Secades-Villa et al., Morasco et al. and Bhattacharjee et al. [5,6,13].

For the supplementary analysis, the ESdE 2023 adult microdata and accompanying data dictionary were downloaded from the official INE website. Of 21,032 respondents aged ≥15 years, 20,639 aged ≥18 years with positive adult survey weights were retained. Variables included age (EDADa), sex (SEXOa), autonomous community or city of residence (CCAA), educational attainment (NIVEST), annual hospitalisation excluding childbirth or caesarean section (J1), annual emergency-service use (J9) and the adult survey weight (FACTORADULTO). Healthcare-use responses were coded as yes or no according to the official dictionary; no missing or invalid responses were identified for either outcome in the retained sample. Survey weights were normalised to estimate population shares and baseline utilisation within strata. National expenditure was extrapolated using the manuscript’s fixed adult population of 40,594,509 and GD prevalence of 0.63%, thereby separating changes in baseline calibration from changes in national population size.

The original analytical implementation used Stata/SE 16.0 (StataCorp LLC, College Station, TX, USA). For this revision, deterministic results and a newly specified restricted PSA were recomputed in Python 3.12.14 with NumPy 2.3.5. Supplementary File S1 documents the input definitions, equations, deterministic analyses and scenario results. Appendix A reports the restricted PSA assumptions and summary results.

## 3. Results

### 3.1. Population and Prevalence

The reference population for the analysis comprised 40,594,509 adults (aged ≥18 years) residing in Spain during the reference year [28] (Table 3). Applying the most recent nationally representative prevalence estimate of gambling disorder (0.63%) [13], based on DSM-5 diagnostic criteria, yielded an estimated 255,745 adults living with gambling disorder in Spain.

The assumed ±20% relative prevalence scenarios (0.504–0.756%) correspond to approximately 204,596–306,894 adults. These scenarios preserve the adult-population denominator but are not confidence bounds.

### 3.2. Selection of Healthcare Cost Components

Two components were costed: emergency department attendance and hospitalisation. Selection decisions and exclusions are reported in Table 1. Primary care and gambling-specific treatment were not implemented quantitatively because suitable excess-use or costing inputs were unavailable; they remain potential extensions.

### 3.3. Base-Case Analysis

Emergency department use contributed EUR 26.20 per adult with GD and EUR 6.70 million nationally. Hospitalisation contributed EUR 1327.97 per adult and EUR 339.62 million nationally. These are excess-cost population averages under the transport and one-episode assumptions, rather than the costs incurred by every service user.

The updated combined annual estimate was EUR 1354.17 per adult with GD and EUR 346.32 million nationally. Hospitalisation accounted for 98.1% of this selected two-service expenditure (Table 4).

### 3.4. Structural Scenarios and Potential Extensions

The following additional scenarios were calculated for this revision to examine the implications of specific assumptions. They are illustrative analyses rather than empirical estimates of alternative Spanish utilisation pathways.

Substituting the NESARC inpatient OR of 1.40 produced EUR 44.60 million nationally; varying this alternative OR over 0.85–2.29 produced −EUR 8.05 million to EUR 122.13 million. The negative lower result reflects a modelled association below one and is not truncated to zero. This scenario demonstrates source dependence and does not validate the alternative as a Spanish causal effect.

#### 3.4.1. Episode Intensity and Emergency Overlap

Multiplying the assumed excess episode count by 1.5 or 2 for both services produced EUR 519.48 million and EUR 692.65 million, respectively. Removing the entire costed emergency component reduced the combined estimate to EUR 339.62 million, a reduction of EUR 6.70 million (1.9%). The latter is the maximum reduction from omitting this component alone, rather than a bound on total model bias.

#### 3.4.2. Potential Service Extensions

Primary care and publicly funded gambling-specific treatment are potential model extensions. No numerical scenario for either service was implemented, so no result or additional expenditure estimate is claimed.

### 3.5. Sensitivity Analyses

One-way results are shown in Table 5. The retained inpatient OR was the largest driver of parameter uncertainty: its confidence limits produced EUR 109.67–EUR 691.76 million nationally. Assumed prevalence variation produced EUR 277.06–EUR 415.59 million; hospital cost variation produced EUR 278.40–EUR 414.25 million. Varying the inpatient baseline by ±20% produced EUR 296.10–EUR 390.11 million, while emergency baseline variation produced EUR 345.52–EUR 346.91 million. These baseline exercises quantify sensitivity but do not correct the age mismatch or comparator differences.

In the restricted PSA, mean national expenditure was EUR 360.90 million, median expenditure was EUR 346.55 million and the conditional 95% simulation interval was EUR 109.12–EUR 689.76 million (Appendix A, Table A1). Only OR uncertainty is propagated; the interval is not a comprehensive uncertainty bound.

**Table 5:** Results of one-way deterministic sensitivity analyses ^1^.

Parameter Varied	Lower Estimate (EUR Million)	Base Case (EUR Million)	Upper Estimate (EUR Million)	Maximum Absolute Effect (EUR Million)	Range Basis
Hospitalisation OR	109.67	346.32	691.76	345.43	Published CI
GD prevalence	277.06	346.32	415.59	69.26	Assumed ±20%
Hospitalisation unit cost	278.40	346.32	414.25	67.92	Assumed ±20%
Hospitalisation baseline probability	296.10	346.32	390.11	50.23	Assumed ±20%
Emergency department OR	341.75	346.32	351.45	5.13	Published CI
Emergency department unit cost	344.98	346.32	347.66	1.34	Assumed ±20%
Emergency department baseline probability	345.52	346.32	346.91	0.80	Assumed ±20%

^1^ Each input was varied independently. OR ranges use published 95% confidence intervals; baseline probabilities, costs and prevalence use assumed ±20% ranges. Those assumptions are neither sampling intervals nor calibrated corrections for age or geographical differences. Maximum absolute effect is the greatest absolute deviation from the updated base case.

### 3.6. Demographic and Regional Sensitivity Analysis

Among 20,639 respondents aged ≥18 years, the weighted annual probabilities of emergency-service use and hospitalisation were 22.74% and 6.96%, respectively. Substituting these adult-specific aggregate probabilities into the original model yielded an annual national estimate of EUR 351.68 million, compared with EUR 346.32 million in the original calculation. Applying the model separately within region–age–sex strata and aggregating the resulting costs yielded EUR 324.85 million annually (EUR 1270.23 per adult with GD), representing a 6.20% reduction relative to the original estimate. Emergency services contributed EUR 6.38 million and hospitalisation EUR 318.47 million. The age–sex and age–sex–education scenarios yielded EUR 332.10 million and EUR 329.49 million, respectively, corresponding to reductions of 4.11% and 4.86%. These were deterministic sensitivity comparisons; no new confidence intervals or significance tests were calculated. The regional analysis included strata with as few as 16 respondents, and six strata had no observed hospitalisations.

## 4. Discussion

This study models selected annual excess emergency and inpatient expenditure associated with GD in Spain. The updated base-case estimate was EUR 346.32 million, or EUR 1354.17 per adult with GD; hospitalisation accounted for 98.1% of this two-service total. These figures are conditional model outputs rather than observed Spanish excess costs, and the analysis makes no claim of national priority or comprehensive healthcare coverage.

The findings suggest that GD is associated with substantial expenditure within the Spanish National Health System. However, the estimates cover selected public healthcare components rather than the total economic burden of gambling-related harm. Although excluding other services may omit relevant expenditure, uncertainty in attribution, healthcare utilisation and potential overlap means that the estimates should not be interpreted as a validated lower bound. Their relevance lies in quantifying a defined component of healthcare expenditure while making the underlying assumptions explicit.

Comparison with studies from other European Union countries provides context for the updated Spanish estimate of EUR 346.32 million annually (EUR 1354 per adult with GD). Differences in case definition, population, included services, attribution methods and price year prevent direct comparison of national totals. Amounts below remain in the original studies’ monetary terms; they have not been harmonised for prices or purchasing power (Table 6).

In Denmark, Vestergaard et al. estimated annual excess direct healthcare costs of EUR 2934 per person with registered GD in 2018 [29], compared with EUR 1354 in the present model. The Danish analysis used linked registries and a clinically identified cohort, whereas the Spanish model extrapolates to all adults estimated to meet GD criteria and includes only emergency and inpatient care. The higher Danish estimate may reflect differences in case selection, resource coverage and costing; it does not establish greater disease severity or healthcare inefficiency in Denmark.

In Sweden, Hofmarcher et al. estimated total societal costs of problem gambling of EUR 1419 million in 2018, including EUR 184 million in direct costs [9]. Importantly, the direct-cost category also included non-healthcare items and is not equivalent to the public healthcare expenditure estimated here. Its estimated EUR 67.29 million for treatment of mental illness provides a healthcare-related comparison, although the underlying attribution assumptions and treatment coverage differ from this model.

More recent Finnish evidence reinforces the sensitivity of cost estimates to methodology. Latvala et al. (2025) reported estimates ranging from EUR 18.28 million to EUR 127.04 million for productivity losses associated with long-term work disability, depending on the attribution method [10]. These are indirect costs and should not be compared numerically with the Spanish healthcare total. Together with the 2025 scoping review [11], this evidence supports transparent reporting of attribution assumptions and cautions against interpreting cross-country differences as differences in the underlying burden alone.

The selection of healthcare components prioritised explicit service definitions and the avoidance of overlapping expenditure. Emergency department utilisation and hospitalisation were included, whereas psychiatric outpatient care, psychological treatment, medication and self-harm-related care were not costed separately because of insufficient evidence on annual excess utilisation or potential overlap with included services. Their exclusion does not imply an absence of associated costs. Some of these resources may already be captured within emergency or inpatient expenditure, while others would require additional utilisation and costing data before they could be incorporated. Future extensions should therefore assess healthcare pathways jointly rather than add each potentially relevant component independently.

The predominance of hospitalisation reflects the combination of the selected association with GD, the baseline probability of admission and the relatively high cost per hospitalisation. Emergency and inpatient care may encompass the assessment and treatment of acute mental-health crises, substance-related problems or co-occurring physical conditions. These examples illustrate possible clinical pathways, but the aggregate utilisation data do not establish which diagnoses account for the estimated excess expenditure. Psychiatric comorbidity and shared socioeconomic or behavioural risk factors may contribute to the observed associations. Consequently, the estimated expenditure cannot be interpreted as entirely caused by GD or as expenditure that would necessarily be prevented through GD treatment alone.

The dominance of inpatient costs is produced jointly by the retained OR, baseline probability, unit cost and exclusion of other services; it does not independently validate the model. Its wide OR interval yields substantial variation, while the alternative inpatient source scenario reduces the total to EUR 44.60 million. The restricted PSA quantifies OR uncertainty only and cannot resolve diagnostic, comparator, age, financing or outcome transportability. These results support cautious interpretation rather than a claim of model robustness.

A strength of the study is the explicit separation of healthcare component selection, utilisation estimates and resource-level cost inputs. The cost per emergency attendance and the mean cost per hospitalisation are inputs to the calculation, whereas the annual excess cost per adult with GD is an estimated population average. Distinguishing these quantities improves the interpretability of the findings and facilitates scrutiny of the calculation. The framework also allows individual inputs and inclusion decisions to be updated as more suitable evidence becomes available.

Several limitations affect the interpretation of these estimates. First, international relative odds do not necessarily transfer between DSM-IV pathological or problem gambling and DSM-5 GD, or between their source comparator groups and the Spanish general population. The inpatient outcome’s recall period and financing mix were not fully matched. Applying these inputs to an annual Spanish baseline therefore remains an unvalidated transport assumption. Adjusted associations also remain vulnerable to residual confounding. Baseline utilisation is not separately calibrated to public financing, which adds uncertainty when valuing contacts as public healthcare resources.

Second, the baseline age mismatch enters the probability conversion directly. The ±20% baseline analyses demonstrate that this can materially influence inpatient expenditure, but do not identify the true difference between ages ≥15 and ≥18. Third, the one-episode calculation can miss repeat contacts; its direction of bias in excess cost depends on recurrence in both comparison groups. The intensity scenarios illustrate this dependence without estimating repeat use. Overlap can act in the opposite direction by counting resources in both components, although complete omission of the modelled emergency component changes the current total by only EUR 6.70 million.

Fourth, the emergency tariff is a regional reimbursement proxy, whereas the hospital input is a provisional national mean that may not reflect the GD case mix. The updated hospital estimate is for 2024; the emergency tariff was introduced in December 2023 and was retained for the reference calculation without an additional inflation adjustment. The two inputs are not a fully harmonised set of national resource costs. Exclusion of services may omit expenditure, but transport assumptions, residual confounding and overlap can bias estimates in either direction, so the result is not a validated lower bound.

The supplementary microdata analysis showed that incorporating regional, age and sex differences in baseline utilisation reduced the national estimate by 6.20%. This quantifies sensitivity to population composition but does not establish full demographic adjustment or validate the international associations used in the model. Constant GD prevalence and common ORs were assumed across strata, so the implied distribution of GD followed the general adult household population rather than an observed GD-specific distribution. Some regional strata were small, and six had no observed hospitalisations, making the regional estimate exploratory. The education scenario was analysed separately and did not jointly incorporate region. Furthermore, the survey excludes institutional residents, and its emergency-use measure covers emergency services generally rather than specifically non-admitted public hospital emergency attendance. Uncertainty concerning residual confounding, financing, regional costs and transportability therefore remains.

Fifth, no comparable adult-prevalence uncertainty bounds were established. The revised PSA therefore fixes prevalence at 0.63%, and its deterministic ±20% range is an assumption. The 1.05% estimate among past-year gamblers is not used as uncertainty for all adults. Fixing other inputs in the restricted PSA improves transparency but means that its interval omits uncertainty in prevalence, baselines, costs and structural assumptions.

The restriction to public healthcare expenditure excludes productivity losses associated with absenteeism, reduced performance at work and reduced employment. Incorporating these consequences could increase the estimated burden from a societal perspective, but would require evidence on their magnitude and attribution to GD. Particular care would be needed to account for psychiatric comorbidity and to avoid counting the same consequences through overlapping measures of lost earnings, lost production or health-related harm [9,30].

The fiscal implications are related but distinct. Reduced employment and earnings may affect tax receipts and public benefits, while gambling also generates revenue. Prior reports examine such wider financial flows [6,31]. Our interpretation is that subtracting gambling tax receipts from this selected healthcare estimate would not constitute a net fiscal assessment: such an assessment would require consistent revenue and expenditure coverage and an explicit counterfactual, including the redirection of gambling spending. This is an interpretation of the analytical scope, rather than a methodological rule established by those references.

These findings may inform healthcare planning and the identification of evidence gaps relevant to prevention, early detection and treatment. They are also consistent with the recognition that gambling-related harm extends beyond individual clinical care and requires a broader public health response [1]. However, a cost-of-illness analysis does not establish the effectiveness or cost-effectiveness of particular interventions. Estimating achievable healthcare savings would require evidence on intervention effects, implementation costs, and the extent to which associated healthcare utilisation can be reduced.

Future research should prioritise longitudinal Spanish evidence on healthcare utilisation among adults with GD, including repeated contacts, reasons for admission and the contribution of psychiatric comorbidity. Better information on the financing and intensity of gambling-specific treatment, together with consistently dated healthcare cost inputs, would support more comprehensive expenditure estimates. These developments would also strengthen subsequent societal and fiscal analyses while preserving clear distinctions between healthcare costs, productivity losses and public revenue effects.

## 5. Conclusions

Under the stated assumptions, the model estimates EUR 346.32 million in annual excess emergency and inpatient expenditure associated with GD in Spain, equivalent to EUR 1354.17 per adult with GD. Hospitalisation contributes 98.1% of this selected two-service total. Source choice and transport assumptions materially affect the result, and the estimate is neither causally attributable expenditure nor a validated lower bound. Spanish evidence with matched diagnostic definitions, comparator groups, annual episode counts and public cost inputs is needed to improve these estimates. No conclusion about intervention effectiveness, avoidable admissions or achievable savings follows from this analysis.

## Figures and Tables

**Table 1:** Selection of candidate cost components.

Cost Component	Association with GD	Meets Inclusion Criteria	Final Classification	Reason
Emergency department	✔	✔	Included in base case	Use association and Spanish tariff; overlap remains uncertain
Hospitalisation	✔	✔	Included in base case	Use association and Spanish cost; transport assumptions required
Primary care	✔	Partial	Potential extension	Limited evidence on annual excess use
Gambling-specific treatment	✔	Partial	Potential extension	Limited national costing evidence
Psychiatry	✔	✘	Excluded	Insufficient excess-use evidence; possible overlap
Psychology	✔	✘	Excluded	Overlap with specialised mental healthcare
Medication	Partial	✘	Excluded	Excess prescribing could not be established
Self-harm-related care	✔	✘	Excluded	High risk of double counting with emergency and inpatient care
Criminal justice	✔	✘	Excluded	Outside the predefined healthcare calculation
Productivity losses	✔	✘	Excluded	Outside the public healthcare perspective
Informal care	✔	✘	Excluded	Outside the public healthcare perspective
Intangible costs	✔	✘	Excluded	Outside the public healthcare perspective

**Table 2:** Principal inputs for deterministic sensitivity analyses ^1^.

Parameter	Base-Case Value	Range Explored	Source
GD prevalence in all adults	0.63%	0.504–0.756% (assumed ±20%)	Spanish adult survey [13]
Emergency department OR	1.98	1.27–3.09	Morasco et al. [5]
Hospitalisation OR	5.575	2.14–14.50	Bhattacharjee et al. [6]
Emergency baseline probability	0.23	0.184–0.276	Spanish aggregate proxy [23]
Hospitalisation baseline probability	0.068	0.0544–0.0816	Spanish aggregate proxy [23]
Emergency tariff per attendance (EUR)	185	148–222	Madrid tariff [27]
Hospitalisation cost per discharge (EUR)	6004.93	4803.944–7205.916	Provisional 2024 SNS mean [26]

^1^ OR, odds ratio. Only OR ranges are published confidence intervals. All ±20% ranges are deterministic assumptions. Baseline age coverage (≥15 years) differs from the target population (≥18 years). The emergency tariff was introduced in December 2023 and retained for the 2024 reference calculation; hospitalisation uses the provisional 2024 national cost. The PSA fixes prevalence, baselines and costs.

**Table 3:** Population parameters used in the model.

Parameter	Value
Adult population in Spain (≥18 years)	40,594,509
GD prevalence in all adults	0.63%
Estimated adults with GD	255,745

**Table 4:** Base-case estimates of annual excess emergency and inpatient expenditure associated with GD in Spain^1^.

Healthcare Component	Cost Per Adult (EUR)	National Cost (EUR Million)	Contribution (%)
Emergency department	26.20	6.70	1.9
Hospitalisation	1327.97	339.62	98.1
Two-service total	1354.17	346.32	100.0

^1^ The 2024 reference calculation combines the provisional national hospital cost and the Madrid tariff introduced in December 2023. Per-adult estimates average over all adults with GD, including non-users. Calculations retain unrounded inputs; displayed values are rounded.

**Table 6:** Contextual comparison with selected international cost-of-illness studies.

Study	Country	Perspective	Main Cost Domains	Direct Comparison Possible	Main Finding
Thorley et al. [7]	UK	Societal	Healthcare, welfare, crime, productivity	No	Broader societal perspective
Public Health England [8]	UK	Public sector	Healthcare, welfare, criminal justice	No direct total comparison	Healthcare represents only one component
Hofmarcher et al. [9]	Sweden	Societal	Healthcare, productivity, crime, family	No	Large indirect costs
Present study	Spain	Public healthcare	Emergency department, hospitalisation	—	Hospitalisation accounts for 98.1% of the two-service model

## Data Availability

Published aggregate inputs are accessible through the cited articles and the INE, Comunidad de Madrid and Ministry of Health sources. No primary participant data were collected or individual-level source records accessed. Supplementary File S1 contains search documentation and source links, exact aggregate inputs, deterministic equations, deterministic results and scenario analyses. Appendix A specifies the restricted PSA assumptions and summary results. Supplementary File S1 does not contain executable scripts or the file of individual simulation draws. Access to underlying source datasets remains governed by their custodians; no public repository for the analytical files is claimed.

## Supplementary Materials

The following supporting information can be downloaded at: https://www.mdpi.com/article/10.3390/healthcare14213685/s1, Supplementary File S1: Search documentation and reproducible expenditure model.

## Abbreviations

The following abbreviations are used in this manuscript:

Abbreviation	Definition
CHEERS	Consolidated Health Economic Evaluation Reporting Standards
CI	Confidence interval
DSM-5	Diagnostic and Statistical Manual of Mental Disorders, Fifth Edition
GD	Gambling disorder
OR	Odds ratio
PSA	Probabilistic sensitivity analysis
SNS	Spanish National Health System (*Sistema Nacional de Salud*)
STROBE	Strengthening the Reporting of Observational Studies in Epidemiology

## Appendix A

The revised restricted PSA used 10,000 iterations with independent log-normal OR distributions. For each service, log(OR) was normally distributed with mean equal to the logarithm of its point estimate and standard deviation [log(upper 95% CI)−log(lower 95% CI)]/(2 × 1.96). The log-scale standard deviations were 0.226825 for emergency use and 0.488098 for inpatient use. Point estimates are therefore distribution medians, not arithmetic means. Both ORs were sampled independently because no cross-study covariance estimate was available; independence is an assumption, not an empirical finding.

Population, prevalence (0.63%), baseline probabilities and unit costs were held fixed. Beta and gamma distributions were not fitted, because their empirical parameterisation was not established; the ±20% deterministic ranges were not treated as 95% intervals. No triangular prevalence distribution was used. Probabilities and costs were calculated at each draw using the same model equations, without truncating negative excess costs. Python 3.12.14 and NumPy 2.3.5 with PCG64 seed 20261020 generated the reported draws. Percentiles use linear interpolation and standard deviations use n − 1. The mean differs from the deterministic result because the OR distributions are skewed and the probability transformation is nonlinear. The resulting interval describes conditional OR uncertainty only.

**Table A1:** Revised restricted probabilistic sensitivity analysis ^1^.

Outcome	Deterministic Estimate	Mean (PSA)	Median (PSA)	Standard Deviation	Conditional 95% Interval
Annual cost per adult with GD (EUR)	1354.17	1411.18	1355.07	594.24	426.66–2697.06
Annual national expenditure (EUR million)	346.32	360.90	346.55	151.97	109.12–689.76

^1^ GD, gambling disorder; PSA, probabilistic sensitivity analysis. Only ORs vary. The national mean is the per-adult mean multiplied by the fixed estimated GD population, apart from displayed rounding. These revised results supersede the earlier PSA and do not retain its prevalence-distribution assumptions.
