# Supplementary material for: Modelling Excess Public Expenditure on Emergency Department and Inpatient Care Associated with Gambling Disorder in Spain
Source: Healthcare (Basel). 2026 Oct 31;14(21):3685. doi: 10.3390/healthcare14213685 (PMC13762612; doi:10.3390/healthcare14213685)
Supplement: Supplementary file 1 [file healthcare-14-03685-s001.zip › healthcare-4622221-supplementary.pdf]

Modelling Excess Public Expenditure on Emergency Department and Inpatient Care Associated with Gambling Disorder in Spain

SUPPLEMENTARY FILE S1: Search documentation and reproducible expenditure model

Targeted evidence assessment

The manuscript describes a targeted, non-systematic assessment dated 01 August 2026, covering PubMed-indexed records, publisher pages, the NIESR report, reference lists and the INE, Ministry of Health and Comunidad de Madrid portals. Its purpose was to verify inputs and identify alternatives. No systematic-review flow diagram, screening counts or claim of exhaustive coverage is made.

The combinations below operationalise the terms described in the manuscript. They are reconstructed, rerunnable search expressions rather than a preserved export or verbatim historical search log. The date above is the date reported by the manuscript, not a claim that these exact strings were executed on that date.

Route	Reconstructed query
**PubMed or publisher search**	("gambling" OR "gambling disorder" OR "pathological gambling" OR "problem gambling") AND ("healthcare utilisation" OR "healthcare utilization" OR "medical utilization" OR hospitalisation OR hospitalization)
**Economic evidence**	(gambling OR "gambling disorder") AND ("cost of illness" OR "cost-of-illness" OR "healthcare costs") AND Spain
**NIESR**	"Fiscal Costs and Benefits of Problem Gambling"; gambling AND inpatient AND APMS
**INE**	"Encuesta de Salud de España 2023" urgencias hospitalización; "población residente" edad 2024
**Ministry of Health**	"Pesos y Costes del SNS 2024" "6.004,93"
**Comunidad de Madrid**	"Orden 1975/2023" urgencias "185"

Eligible utilisation evidence concerned adults, identifiable healthcare contacts and an extractable adjusted association with uncertainty bounds. Where multiple models were reported, demographic and behavioural or clinical adjustment was preferred. Statistical significance was not an eligibility criterion. Associations with different definitions and comparators were not pooled. Primary care and gambling-specific treatment were not quantified.

Input definitions and source links

Parameter	Exact value	Manuscript source
**Adult resident population**	40,594,509	INE [28]
**GD prevalence among all adults**	0.0063	Secades-Villa et al. [13]
**Emergency baseline probability**	0.23	INE ESdE [23]
**Inpatient baseline probability**	0.068	INE ESdE [23]

**Emergency adjusted OR and 95% CI**	1.98 [1.27, 3.09]	Morasco et al. [5]
**Inpatient adjusted OR and 95% CI**	5.575 [2.14, 14.50]	Bhattacharjee et al. [6]
**Alternative inpatient OR and 95% CI**	1.40 [0.85, 2.29]	Morasco et al. [5]
**Emergency tariff per attendance**	EUR 185	Madrid [27]
**Inpatient cost per discharge**	EUR 6,004.93	Ministry of Health [26]

Source numbering below follows the manuscript. Links identify the cited sources; retaining a model input is distinct from independently validating its extraction or transferability.

[5] Morasco, B.J.; Pietrzak, R.H.; Blanco, C.; Grant, B.F.; Hasin, D.; Petry, N.M. Health problems and medical utilization associated with gambling disorders: results from the National Epidemiologic Survey on Alcohol and Related Conditions. Psychosom. Med. 2006, 68, 976–984. https://doi.org/10.1097/01.psy.0000238466.76172.cd.

[6] Bhattacharjee, A.; Dolton, P.; Mosley, M.; Pabst, A. The Fiscal Costs and Benefits of Problem Gambling: Towards Better Estimates; National Institute of Economic and Social Research: London, UK, 2023. Available online: https://niesr.ac.uk/publications/fiscal-costs-benefits-problem-gambling

[13] Secades-Villa, R.; Krotter, A.; Aonso-Diego, G. Prevalence and correlates of gambling disorder in Spain: findings from a national survey. Int. Gambl. Stud. 2024, 24, 325–340. https://doi.org/10.1080/14459795.2023.2276747.

[23] Instituto Nacional de Estadística. Encuesta de Salud de España (ESdE). Año 2023. Available online: https://www.ine.es/dyngs/Prensa/ESdE2023.htm

[24] Instituto Nacional de Estadística. Metodología Encuesta de Salud de España 2023; INE: Madrid, Spain, 2025. Available online: https://ine.es/metodologia/t15/esde23_meto.pdf

[25] National Centre for Social Research. Adult Psychiatric Morbidity Survey 2007 Interviewer Instructions; UK Data Service, Study 6379. Available online: https://doc.ukdataservice.ac.uk/doc/6379/mrdoc/pdf/6379_apms_2007_interviewer_instructions.pdf

[26] Ministerio de Sanidad, Subdirección General de Información Sanitaria. Proceso de Estimación de Pesos y Costes Hospitalarios del SNS: Pesos y Costes del SNS 2024 (Provisionales); Ministerio de Sanidad: Madrid, Spain, 2026. Available online: https://www.sanidad.gob.es/estadEstudios/estadisticas/docs/CMBD/2024_nota_metodologica_costes.pdf

[27] Comunidad de Madrid. Orden 1975/2023, de 29 de diciembre, de la Consejera de Sanidad, por la que se fijan los precios públicos por la prestación de los servicios y actividades de naturaleza sanitaria de la Comunidad de Madrid. Bol. Of. Comunidad Madr. 2023, 310. Available online: https://www.comunidad.madrid/transparencia/sites/default/files/bocm-20231230-3.pdf

[28] Instituto Nacional de Estadística. Estadística Continua de Población: Población residente por fecha, sexo y edad (desde 1971). Available online: https://ine.es/jaxiT3/Tabla.htm?t=56940

The emergency OR concerns lifetime DSM-IV pathological gambling and past-year service use in US NESARC, with a low-risk comparator. The retained inpatient estimate concerns problem gambling in an English APMS-based analysis with a different risk-group counterfactual. These are not equivalent to current DSM-5 GD versus the Spanish general population. The alternative inpatient association comes from the same NESARC study as the emergency OR.

The baseline proxies cover ages 15 years or older, whereas prevalence and national extrapolation cover ages 18 years or older. The inpatient measure excludes childbirth; recall periods, private/public coverage and case mix remain potential sources of mismatch. The emergency tariff is a regional reimbursement proxy introduced in December 2023, retained for the 2024 reference calculation. The inpatient input is the provisional 2024 national acute hospital cost.

Equations and deterministic analyses

For service j, let p0j denote the baseline probability, ORj the adjusted odds ratio and cj the unit cost. The model uses:

pGDj = ORj × p0j / (1 − p0j + ORj × p0j)
Excess probabilityj = pGDj − p0j
Cost per adult with GD = sumj [(pGDj − p0j) × cj]
Estimated GD population = 40,594,509 × 0.0063 = 255,745.4067
National expenditure = cost per adult with GD × estimated GD population

National extrapolation retains the fractional estimated population. Rounding to 255,745 before multiplication would change the exact results. The one-episode approximation values a difference in probabilities of any use; it does not measure recurrent episode counts. No discounting is applied over the one-year horizon.

Component	Per adult EUR	National EUR million	Share percent
**Emergency**	26.20	6.70	1.9
**Inpatient**	1,327.97	339.62	98.1
**Total**	1,354.17	346.32	100.0

One-way analyses vary each OR across its reported 95% confidence interval. Prevalence, baselines and unit costs vary by an assumed ±20% relative change. All other inputs remain fixed. These assumed ranges are not empirical uncertainty intervals.

Varied parameter	Lower national EUR million	Upper national EUR million
**or_ip**	109.67	691.76
**prevalence**	277.06	415.59
**cost_ip**	278.40	414.25
**p_ip**	296.10	390.11
**or_ed**	341.75	351.45
**cost_ed**	344.98	347.66
**p_ed**	345.52	346.91

Structural scenarios replace the inpatient OR with 1.40 and its limits of 0.85 and 2.29, multiply both excess episode proxies by 1.5 or 2, or omit all costed emergency expenditure. Negative excess costs are retained.

Scenario	Per adult EUR	National EUR million

**base**	1,354.17	346.32
**alternative_inpatient_low**	-31.47	-8.05
**alternative_inpatient**	174.40	44.60
**alternative_inpatient_high**	477.54	122.13
**intensity_1_5**	2,031.26	519.48
**intensity_2**	2,708.34	692.65
**omit_emergency**	1,327.97	339.62
